# Redefining differential roles of MAO-A in dopamine degradation and MAO-B in tonic GABA synthesis

**DOI:** 10.1038/s12276-021-00646-3

**Published:** 2021-07-09

**Authors:** Hyun-U Cho, Sunpil Kim, Jeongeun Sim, Seulkee Yang, Heeyoung An, Min-Ho Nam, Dong-Pyo Jang, C. Justin Lee

**Affiliations:** 1 Department of Biomedical Engineering, Hanyang University, Seoul, Korea; 2 KU-KIST Graduate School of Converging Science and Technology, Korea University, Seoul, Korea; 3 Center for Cognition and Sociality, Institute for Basic Science, Daejeon, Korea; 4 Center for Neuroscience, Brain Science Institute, Korea Institute of Science and Technology (KIST), Seoul, Korea; 5 Department of KHU-KIST Convergence Science and Technology, Kyung Hee University, Seoul, Korea

**Keywords:** Neurotransmitters, Biosensors, Astrocyte, Inhibition, Transporters in the nervous system

## Abstract

Monoamine oxidase (MAO) is believed to mediate the degradation of monoamine neurotransmitters, including dopamine, in the brain. Between the two types of MAO, MAO-B has been believed to be involved in dopamine degradation, which supports the idea that the therapeutic efficacy of MAO-B inhibitors in Parkinson’s disease can be attributed to an increase in extracellular dopamine concentration. However, this belief has been controversial. Here, by utilizing in vivo phasic and basal electrochemical monitoring of extracellular dopamine with fast-scan cyclic voltammetry and multiple-cyclic square wave voltammetry and ex vivo fluorescence imaging of dopamine with GRAB_DA2m_, we demonstrate that MAO-A, but not MAO-B, mainly contributes to striatal dopamine degradation. In contrast, our whole-cell patch-clamp results demonstrated that MAO-B, but not MAO-A, was responsible for astrocytic GABA-mediated tonic inhibitory currents in the rat striatum. We conclude that, in contrast to the traditional belief, MAO-A and MAO-B have profoundly different roles: MAO-A regulates dopamine levels, whereas MAO-B controls tonic GABA levels.

## Parkinson’s disease: rewriting the roles of a critical enzyme

The inhibition of two forms of an enzyme that modulate key processes in the brain has different benefits for patients with Parkinson’s disease than previously thought. Monoamine oxidase (MAO) is present in the brain as MAO-A and MAO-B, both of which were thought to be involved in dopamine degradation. MAO inhibitors are used to limit dopamine degradation in Parkinson’s disease and depression, improving symptoms by increasing levels of usable dopamine. In experiments on rats, Hyun-U Cho at Hanyang University, Seoul, South Korea, and coworkers have shown that MAO-A, but not MAO-B, affects dopamine degradation. The team found that MAO-B instead mediates the synthesis of a key neurotransmitter, GABA, the upregulation of which is linked to Parkinson’s motor symptoms. Taking MAO-B inhibitors may be addressing these symptoms, explaining why patients show improvement.

## Introduction

Monoamine oxidase (MAO) is an enzyme that catalyzes the oxidative deamination of biogenic amines^1,2^. MAOs are localized in the outer membrane of mitochondria and have an important role in the metabolism of neuroactive monoamines, such as dopamine (DA), norepinephrine, serotonin, and melatonin, in the brain^3^. MAO exists as two isoenzymes, MAO-A and MAO-B, with different substrate specificities^4^. In particular, epinephrine, norepinephrine, melatonin, and serotonin are mainly metabolized by MAO-A, whereas phenylethylamine and benzylamine are mainly degraded by MAO-B^5^. The two isozymes also have differential cellular localizations in the brain: MAO-A is predominantly localized at the nigrostriatal DAergic axon terminals, while MAO-B is mostly localized in astrocytes and serotonergic neurons^6–8^. Owing to their substrate specificities, selective MAO inhibitors have been designed to inhibit the activity of a specific type of isoenzyme: clorgiline and moclobemide inhibit MAO-A, while selegiline, rasagiline, safinamide, and KDS2010 inhibit MAO-B^9–12^. Most of these MAO-A and MAO-B inhibitors are differentially prescribed to patients with depression and Parkinson’s disease (PD), respectively^9^.

Among various monoamine neurotransmitters, DA has traditionally been thought to be degraded by both MAO-A and MAO-B^13–17^. Accumulated DA in the synaptic cleft is taken up by DA neurons and surrounding astrocytes and then degraded by MAO and catechol-O methyltransferase (COMT). However, the contribution of MAO-B to DA degradation has been controversial. For instance, it has been demonstrated that MAO-B-deficient mice show no difference in striatal DA levels compared with wild-type mice^18^. Moreover, acute treatment with selegiline, an MAO-B inhibitor, has been reported to be ineffective in altering the striatal efflux of DA, whereas acute treatment with clorgiline, an MAO-A inhibitor, reduces DA metabolism^19–21^. Pargyline, another MAO-B inhibitor, was also reported to be ineffective in altering evoked DA release in the nucleus accumbens^22^. Furthermore, it has been previously reported that MAO-B inhibition shows only a marginal effect of blocking DA degradation in rat models of PD, while MAO-A inhibition shows a dramatic effect^23^. Although these accumulating lines of evidence have cast doubt on the major role of MAO-B in DA degradation, MAO-B is still widely believed to be involved in DA degradation due to the positive therapeutic effect of MAO-B inhibitors in PD patients.

In a series of recent studies, we demonstrated that the therapeutic effect of MAO-B inhibitors in PD could be mainly attributed to a decrease in the tonic inhibition of DA neurons in the SNpc^24^, based on compelling lines of evidence that MAO-B is the critical enzyme for GABA synthesis in reactive astrocytes^24–27^. We determined that MAO-B mediates astrocytic GABA synthesis through the putrescine degradation pathway in various brain areas, including the hippocampus, cerebellum, striatum, cortex, and SNpc^11,24–27^. Astrocytic GABA can be tonically released through a Ca^2+^-activated anion channel, Best1^25,28,29^. Astrocytically released GABA binds to extrasynaptic GABA receptors to tonically inhibit the activities of neighboring neurons^28^. In addition, when astrocytes become reactive upon various physical or chemical insults, MAO-B-mediated astrocytic GABA synthesis is aberrantly upregulated^24,25,30,31^, leading to various neurological symptoms, such as parkinsonian motor symptoms in PD. In contrast to MAO-B, the possible role of MAO-A in tonic GABA inhibition has not been rigorously tested.

In this study, we investigated the contributions of MAO-A and MAO-B to nigrostriatal DA degradation and tonic GABA inhibition by multifaceted approaches, including in vivo fast-scan cyclic voltammetry (FSCV)^32^, in vivo multiple-cyclic square wave voltammetry (M-CSWV)^33^, ex vivo DA imaging^34^, and ex vivo whole-cell patch-clamp recording. Based on these findings, we redefined the differential roles of MAO-A and MAO-B: MAO-A participates in DA degradation and MAO-B in tonic GABA synthesis.

## Materials and methods

### Animals

Adult male Sprague Dawley rats (Koatech, Korea) weighing 250–350 grams (8 to 13 weeks old) were used for in vivo experiments for FSCV and M-CSWV recording (*n* = 7, each group). Male and female adult C57BL/6J mice (IBS Research Solution Center; 8 to 16 weeks old) were used for ex vivo DA imaging. Adult male Sprague Dawley rats (Daehan Biolink, Korea) weighing between 200–250 g (6 to 8 weeks old) were used for ex vivo patch-clamp experiments for tonic GABA recording (*n* = 5, each group). The animal experimental procedures were approved by the Institutional Animal Care and Use Committee of Hanyang University (Seoul, Korea), Korea Institute of Science and Technology (KIST; Seoul, Korea), and Institute for Basic Science (IBS; Daejeon, Korea). The animals were kept on a 12 h light-dark cycle with controlled temperature and humidity and had ad libitum access to food and water. Animal care was performed following National Institutes of Health (NIH) guidelines.

### Chemicals

For pharmacological tests, selegiline and clorgiline were chosen for selective MAO-A and MAO-B inhibition and purchased from Sigma-Aldrich (St. Louis, MO). The reversible MAO-B inhibitor KDS2010 was provided by KIST. For in vivo FSCV and M-CSWV, selegiline (10 mg kg^−1^; i.p.), clorgiline (10 mg kg^−1^; i.p.) or KDS2010 (10 mg kg^−1^; i.p.) was dissolved in DI water and injected as a single dose of 500 µL. For ex vivo DA imaging, each compound (100 nM) was applied to the bath. For ex vivo patch-clamp experiments, acute brain slices were pre-incubated in each compound (100 nM).

### In vivo voltammetry

#### Surgery

Prior to surgery, rodents were anesthetized with intraperitoneal urethane (1.6 g/kg, i.p., Sigma-Aldrich, St Louis, MO) and stabilized on a stereotaxic frame (David Kopf Instruments, Tujunga, CA). The body temperature was maintained at 37 °C using a heating pad (TCAT-2, Harvard Apparatus). An incision was made in the skin to expose the skull, and then three holes with diameters of 0.5–1.0 mm were drilled in the skull for the implantation of a carbon fiber microelectrode (CFM), a bipolar electrical stimulating electrode (Plastic One, MS303/2, Roanoke, VA, USA), and an Ag/AgCl reference electrode. The reference electrode was positioned superficially in the cortical tissue contralateral to the hemisphere where the CFM and stimulating electrode were implanted. Electrode coordinates were defined by the rat brain atlas^35^ based on the skull position using the bregma as a reference point with coordinates anteroposterior (AP), mediolateral (ML), and dorsoventral (DV). The CFM was placed in the right hemisphere in the striatum (AP + 1.2 mm; ML + 2.0 mm; DV −4.5 to −5.5 mm), and the stimulating electrode was inserted into the medial forebrain bundle (MFB, AP −4.6; ML + 1.3; DV −8.0 to −9.0).

#### Electrodes

CFMs were fabricated as previously described^33^. A single carbon fiber (AS4, d = 7 μm; Hexel, Dublin, CA) was used for all experiments. The fiber was inserted into silica tubing (20 µm ID, 90 µm OD, 10 µm coated with polyimide; Polymicro Technologies, Phoenix, AZ) and covered with polyamic acid (Sigma-Aldrich, St. Louis, MO) at one end. The electrode was then heated to 200 °C to polymerize the polyamic acid into a polyimide film for fixation. The silica tubing was then attached to a nitinol wire (Nitinol #1, an alloy of nickel and titanium; Fort Wayne Metals, IN) with a commercial silver conductive paste (Sigma-Aldrich, St. Louis, MO). The carbon fiber-attached nitinol wire was insulated with polyimide tubing (0.0089”ID, 0.0134”OD, 0.00225” WT; Vention Medical, Salem, NH) with the carbon side exposed on the exterior. After the silver paste became solid, the exposed carbon fiber was trimmed under a dissecting microscope to a length of approximately 80–100 μm using a scalpel blade. PEDOT:Nafion coating was applied to the exposed carbon fiber by electrical deposition with a mixture of poly(3,4-ethylenedioxythiophene) and Nafion to increase their sensitivity and selectivity to DA and reduce in vivo biofouling^36^. Teflon-coated silver wire (A-M Systems, Inc., Sequim, WA) was prepared as an Ag/AgCl reference electrode.

#### FSCV

Data collection and analysis of FSCV were performed using a commercial electronic interface (NIUSB-6363, National Instruments) with a base-station PC and software written in-house using LabVIEW 2016 (National Instruments, Austin, TX). FSCV was applied with a conventional triangular waveform (– 0.4 V to 1.3 V versus Ag/AgCl at 400 V/s scan rate) applied at a 10 Hz repetition rate. Before recording, the CFM and the electrical stimulating electrode were gradually adjusted until a robust phasic DA signal was detected. A train of bipolar pulses (2 ms pulse width, 350 µA, 60 Hz, 120 pulses) was delivered for 2 s every 10 min to evoke DA release. Before drug treatment, the carbon microelectrode was positioned and stabilized to the right recording site to optimize the DA signal. Phasic DA was evoked by biphasic electric stimulation of the MFB every 10 min. Each animal dataset consisted of a voltammogram of 40 min before and 180 min after treatment with an MAO inhibitor drug. We quantified the DA degradation kinetics with the exponential decay time (rate constant: K), which was fitted to a one-phase exponential curve.

$${\mathrm{Y}}({\mathrm{t}}) = {\mathrm{A}}0^ \ast {\mathrm{e}}^{ - {\mathrm{k}} \ast {\mathrm{t}}} + {\mathrm{B}}0$$

where *t* represents the time, *k* is the decay constant, *A0* is the starting value, and *B0* is the plateau value. Data processing, including background subtraction, averaging, and digital filtering, was performed for all voltammogram datasets by MATLAB (MathWorks Inc., Natick, MA, USA).

#### M-CSWV

M-CSWV was performed using the same equipment used in the FSCV experiment except for an in-house built current-to-voltage amplifier without an analog filter to preserve sharp current responses to the square pulse. M-CSWV data were collected in the form of a sequence of unsigned 2-byte integers, saved to the base-station computer, and processed with a custom software control made by MATLAB (MathWork Inc., Natick, MA). Data processing included temporal averaging, filtering, and simulating background currents. The basal DA response was extracted from the 2-dimensional voltammogram using the DA-kernel method. We also used the same M-CSWV parameters proposed in the previous research^33^. Five cyclic square waveforms, which consisted of a large-amplitude square wave modulation on top of a symmetric staircase waveform, were applied in sequence at a frequency of 0.1 Hz. *E*_Staircase_, *E*_Initial_ (=*E*_End_), and τ(tau) were fixed at 25 mV, −200 mV, and 1.0 ms, respectively. The experimental protocol of basal-level monitoring was performed by the same protocol as the FSCV experiment. Once an optimal CFM recording site was identified, the device was changed to the M-CSWV recording system for tonic DA concentration recording. In contrast to the FSCV experiment, however, rats were constantly measured without electrical stimulation. Pharmacological effects on tonic DA concentrations were calculated in a circular DA area extracted from the maximum amplitude of the pseudocolor plot.

### Ex vivo DA imaging

#### Virus construct

The AAV-hSyn-GRAB_DA2m_ viral vector was kindly provided by Yulong Li’s laboratory. AAV containing hSyn-GFAB_DA2m_ was packaged by the IBS virus facility (Daejeon, Korea). All viruses were diluted immediately before injection (1/5 dilution with saline).

#### Stereotaxic injection

Mice were anesthetized with 1–2% isoflurane and placed on a stereotaxic apparatus (68537, RWD). After incision, the skull was cleaned and leveled in the coronal and sagittal planes using the bregma and lambda as reference points. Using a dental drill, holes were made bilaterally in the skull targeting the striatum using the following coordinates^37^: AP, 0.5 mm; ML, ±1.5 mm; DV, −3.3 mm from the bregma. AAV expressing hSyn-GRAB_DA2m_ was loaded into a vertically pulled VWR microdispenser (53508–375, SP Scientific). Virus injection was performed using a microinfusion pump (Legato 130, KD Scientific) at a rate of 0.1 μL/min with a 0.5 μL volume in each hemisphere. The injection needle was left in place for an additional 10 min before the withdrawal. After at least 2 weeks of recovery, the mice were used for experiments.

#### DA imaging

Animals were deeply anesthetized with 1–2% isoflurane, followed by decapitation. The brain was quickly excised from the skull and submerged in ice-cold dissection buffer (in mM): 212.5 sucrose, 26 NaHCO_3_, 10 D-glucose, 5 MgCl_2_, 3 KCl, 1.25 NaH_2_PO_4_, and 0.1 CaCl_2_; pH 7.4, saturated with 95% O_2_ and 5% CO_2_. Coronal slices of 300-μm thickness were cut with a vibratome (D.S.K LinearSlicer pro 7, Dosaka EM Co. Ltd). Slices were then transferred to an incubation chamber with artificial cerebrospinal fluid (aCSF) (in mM): 130 NaCl, 24 NaHCO_3_, 3.5 KCl, 1.25 NaH_2_PO_4_, 1.5 CaCl_2_, 1.5 MgCl_2_, and 10 glucose; pH 7.4, at room temperature. After 1 h of stabilization, brain slices were transferred into a perfusion chamber where aCSF continuously flowed over slices at 1–2 mL/min. To visualize the GRAB_DA2m_ sensor, a blue LED (pE340^fura^, CoolLED) was applied to brain slices under a fluorescent upright microscope with a ×10 objective. After confirming sensor expression, phasic DA release was evoked by a single pulse (40 µA with a 2 ms pulse width) from the stimulating electrode. Stimulations were administered near the sensor-expressed region every 10 mins during the experiments. After the first electrical stimulation, we chose approximately ten different regions of interest (ROIs) that responded to stimulation. To measure the phasic DA release, the peak of the sensor signal was normalized by its baseline (ΔF/F_0_ ratio). Before drug treatment, the baseline phasic DA release was measured by acquiring 3 successive similar levels of DA signals. It usually took 30–60 mins to obtain stable DA release. After stabilization, drugs were delivered by continuous bath application for 1 h. Every 10 min, the peak DA release was measured in every ROI and averaged to obtain a single value. Acquisition and ROI analysis was performed using Imaging Workbench (INDEC Biosystem) and ImageJ (NIH).

### Whole-cell patch-clamp

Acute 300 μm-thick striatum-containing slices were prepared with a vibratome (D.S. K LinearSlicer pro 7, Dosaka EM Co. Ltd) and washed in aCSF buffer containing (in mM): 134 NaCl; 2.5 KCl; 1.3 MgCl_2_; 2 CaCl_2_; 1.25 K_2_HPO_4_; 26 NaHCO_3_; 10 d-glucose at pH 7.4 for at least 1 h. The buffer solution was saturated with carbogen (95% O2, 5% CO2). The slices were transferred to a recording chamber and constantly washed with aCSF solution at a speed of 2 mL/min. All experiments were performed in voltage-clamp mode. The slice chamber was mounted on the stage of an upright Olympus microscope and visualized with a 60x water immersion objective (NA = 0.90) with infrared differential interference contrast optics and a CCD camera by using Imaging Workbench software (Indec BioSystems). Whole-cell recordings were performed on striatal neurons located in the dorsal striatum. Most of these recorded striatal neurons were medium spiny neurons by morphology. However, we did not further characterize them as D1-positive or D2-positive neurons. The cells were held at −60 mV. The pipette had a resistance of 6–8 MΩ and was filled with the following intracellular solution (in mM): 135 CsCl; 4 NaCl; 0.5 CaCl_2_; 10 HEPES; 5 EGTA; 10 QX-314; pH 7.2 with NaOH (278–285 mOsmol). Before recording the tonic GABA current, the baseline current was stabilized with D-AP5 (50 μM) and CNQX (20 μM). Electrical signals were digitized and sampled at 50 ms intervals with a Digidata 1440 A and a Multiclamp 700B amplifier (Molecular Devices) using pCLAMP 10.2 software. Data were filtered at 2 kHz. The amplitude of the tonic GABA current was measured by the baseline shift after bicuculline (50 μM) administration using Clampfit 10.7 (Molecular Devices). The frequency and amplitude of spontaneous inhibitory postsynaptic currents (sIPSCs) before bicuculline administration were detected and analyzed by Template Search in Clampfit 10.7.

### Statistical analysis

Statistical analyses were performed using Prism 8 (GraphPad Software, Inc.). For the comparison of two groups, an unpaired Student’s *t*-test was used. For the comparison of multiple groups, a one-way analysis of variance (ANOVA) with Tukey’s multiple comparison test was used. For the assessment of differences associated with a certain intervention in two groups, the significance of the data was assessed by paired Student’s *t*-test. Data from multiple independent experiments were assumed to have normal variance. All data were tested to determine whether they were normally distributed. For the data that were not normally distributed, the Kruskal-Wallis test was used instead of one-way ANOVA for statistical analysis. When the data showed unequal variance between groups, Brown-Forsythe and Welch ANOVA tests were used with Dunnett’s multiple comparison test. *P* < 0.05 was considered to indicate statistical significance throughout the study. The significance level is represented as asterisks (*P < 0.05, **P < 0.01, ***P < 0.001; ns, not significant). All data are presented as the mean ± SEM. All raw data are presented as dot plots in each graph. The numbers of animals used are described in the corresponding figure legends or on each graph. Experimental groups were balanced in terms of animal age, sex, and weight. The sample size was determined based on the expected difference from our pilot studies using similar protocols in animals without carrying out a formal power analysis. At least three independent repeats were performed for each experiment. No outliers were excluded.

## Results

### Inhibition of MAO-A, but not MAO-B, augments phasic DA release

**Fig. 1:**
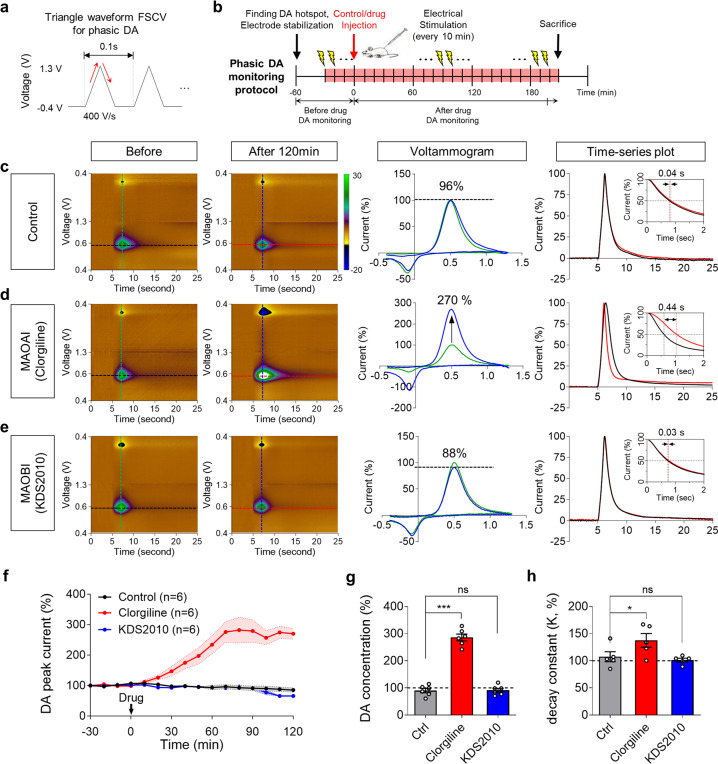
Evoked DA response monitored with FSCV in dorsal striatum show increment during MAO-A inhibition, while less change at MAO-B inhibition. **a** Schematic design of triangular waveform from fast cyclic voltammetry (FSCV). **b** Experimental schedule for FSCV recordings. **c**–**e** The representative phasic dopamine changes in the striatum detected by FSCV in response to treatment with saline control, MAO-A inhibitor (clorgiline, 10 mg kg^−1^), and MAO-B inhibitor (KDS2010, 10 mg kg^−1^). Pseudo-color plots indicate the phasic dopamine responses before (left) and after 120 mins (middle) from drug administration. The time-series plot of each evoked DA response was extracted at the dopamine oxidation potential (right). The black line and red line show the concentration change before and after drug injection, respectively. **f** Time-dependent change in the peak DA current. **g** Quantification of estimated DA concentrations from peak DA current. MAO-A inhibition increased phasic DA level to 284%, while MAO-B inhibition did not (*n* = 6 mice for each group). **h** Summary bar graph of the decay time constant *K* calculated from the time-series plots at the oxidation voltage (0.6 V). Data represent mean ± SEM. *P < 0.05; ***P < 0.001; ns, non-significant by one-way ANOVA with Tukey’s multiple comparison test.

To investigate the roles of MAO-A and MAO-B in the regulation of phasic and basal DA levels, we adopted a pharmacological approach utilizing potent and selective inhibitors against MAO-A (clorgiline) MAO-B (KDS2010^11^ and selegiline). As previously defined^38^, the phasic DA level indicates the evoked synaptic dopamine concentration, while the basal DA level (or tonic DA level) indicates the steady-state level of extrasynaptic dopamine concentration, which is a combination of tonic DA neuronal activity and spontaneous phasic activity. We first tested whether the pharmacological inhibition of the two isoenzymes differentially affected the phasic release of striatal DA by performing in vivo FSCV with electrical stimulation of the MFB of a rat^39^. To monitor the DA current, typical triangular voltage waveforms with a scan rate of 400 V s^−1^ from −0.4 to 1.3 V and with a 10-Hz repetition rate^32^ were given through a carbon microfiber electrode implanted in the dorsal striatum (Fig. 1a, b). We first confirmed that the electrical stimulation of the MFB faithfully evoked an oxidation signal at approximately +0.6 V, which indicated the DA current (Fig. 1c). We also confirmed that vehicle injection did not significantly alter the evoked DA current (90.20 ± 7.89%) (Fig. 1c, f, g). We found that the intraperitoneal administration of clorgiline (10 mg kg^−1^; MAO-A inhibitor) significantly and gradually increased the peak current of the evoked DA release for 120 min (286.1 ± 12.52%), which indicated that MAO-A negatively regulates the peak current of phasic DA (Fig. 1d, f, g). On the other hand, KDS2010 treatment (10 mg kg^−1^; MAO-B inhibitor) did not alter the peak DA current (91.37 ± 7.28%) (Fig. 1e, f, g). Another MAO-B inhibitor, selegiline, showed a similar effect to KDS2010 (Supplementary Fig. 1a-c). These findings suggest that MAO-A, but not MAO-B, could be responsible for the degradation of intracellular DA, which is directly associated with the amount of DA released upon electrical stimulation.

Next, to estimate the change in the rate of extracellular DA clearance upon treatment with MAO-A and MAO-B inhibitors, we analyzed the decay time constant *K* before and after 120 mins of drug treatment from the time-series plots obtained from the DA oxidation voltage (+0.6 V) (Fig. 1c–e). We found that MAO-A inhibition significantly increased the decay time constant *K* (36.67 ± 12.59%) compared to that of the control group (7.50 ± 9.20%), while MAO-B inhibition did not (Fig. 1h and Supplementary Fig. 2). These findings suggest that MAO-A inhibition, but not MAO-B inhibition, significantly retarded DA clearance from the extracellular space. Consistently, a previous report showed that DA clearance is mediated mostly by DA uptake through the DA transporter (DAT), which is expressed mainly by neighboring DAergic neurons but minimally by astrocytes in vivo^40^. Therefore, MAO-A inhibition is likely to hinder DA degradation, which might lead to a delay in extracellular DA uptake, whereas MAO-B is less likely to contribute to DA degradation. Another possibility is that the amount of DA released in clorgyline-treated animals may saturate DAT, thus widening the peak. These possibilities await future investigation.

**Fig. 2:**
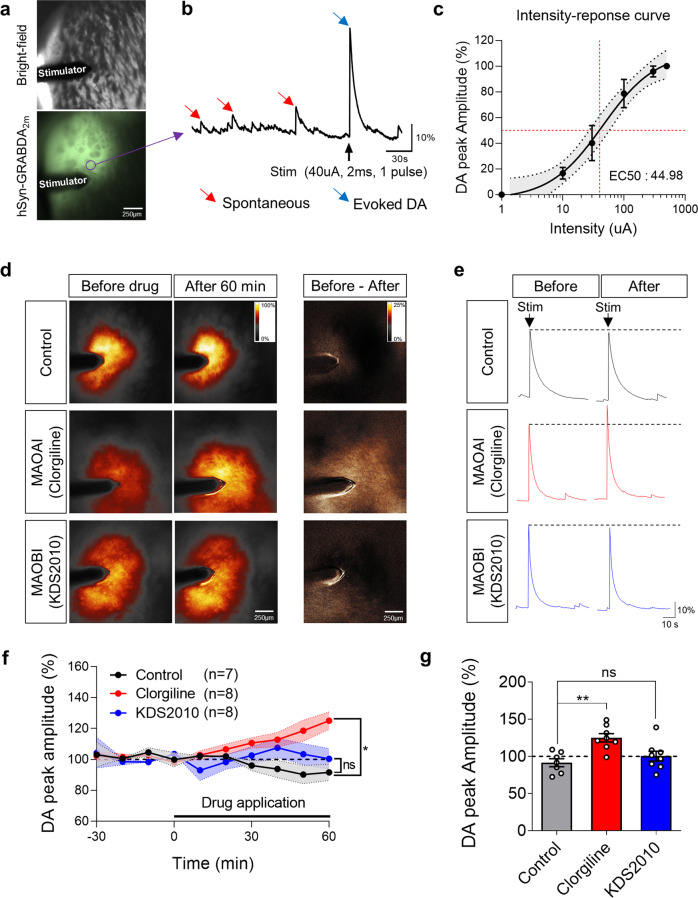
MAO-A inhibitor, but not MAO-B inhibitor, increased phasic DA release in mouse striatum. **a** Representative image of GRAB_DA2m_ expression in mouse striatum. **b** Example DA trace from one ROI (magenta circle in **a**). Both spontaneous (red arrow) and evoked (blue arrow) are monitored by GRAB_DA2m_. **c** Intensity-response curve of GRAB_DA2m_ in mouse striatum (*n* = 4, non-linear regression, EI_50_ = 44.98 pA). **d** Representative images of phasic DA release level in each condition: Control, MAO-A inhibitor (clorgiline, 100 nM), and MAO-B inhibitor (KDS2010, 100 nM). **e** Example DA trace from d. The dotted line indicates the level of phasic DA release before drug treatment. **f** Time-dependent changes in DA peak amplitudes before and during drug treatment (*N* = 7, 8, and 8). Phasic dopamine release is significantly increased by MAO-A inhibition (clogiline), but not by MAO-A inhibition (KDS2010). **g** Summary bar graph of DA peak amplitude 60 mins after the treatment of clorgiline and KDS2010 (*N* = 7, 8, and 8). Data represent mean ± SEM. *P < 0.05; **P < 0.01; ns, non-significant by repeated measures one-way ANOVA with Dunnett’s multiple comparisons test (**f**) or by one-way ANOVA with Tukey’s multiple comparison test (**g**).

To strengthen our observation that MAO-B is less likely to mediate DA degradation, we additionally performed ex vivo DA imaging using a recently developed genetically encoded DA sensor, GRAB_DA2m_^34^. To express GRAB_DA2m_ in striatal neurons, we injected adeno-associated virus (AAV) expressing GRAB_DA2m_ under the human synapsin (hSyn) promoter (AAV-hSyn-GRAB_DA2m_) into the striatum of a mouse (Fig. 2a). The DA responses were sensitively detected by a GRAB_DA2m_ sensor in acute tissue slices of the striatum (Fig. 2b). The amplitude of the electrical stimulation-evoked DA response was dependent on the intensity of the electrical stimulation (Fig. 2c). We chose a 40 μA stimulation intensity, which corresponded to the median effective intensity (EI_50_) for phasic DA release. While the DA signal was recorded, inhibitors against MAO-A or MAO-B were applied to the bath. Consistent with the findings from in vivo FSCV, MAO-A inhibition by 100 nM clorgiline gradually and significantly increased the DA peak amplitude (125.08 ± 5.67%), whereas MAO-B inhibition by 100 nM KDS2010 (100.39 ± 6.73%) or 100 nM selegiline (96.37 ± 1.45%) did not (Fig. 2d–g and Supplementary Fig. 1d–f). These findings underpin our conclusion that the inhibition of MAO-A, but not MAO-B, blocks DA degradation in the mouse striatum.

### Inhibition of MAO-A, but not MAO-B, elevates basal DA levels

**Fig. 3:**
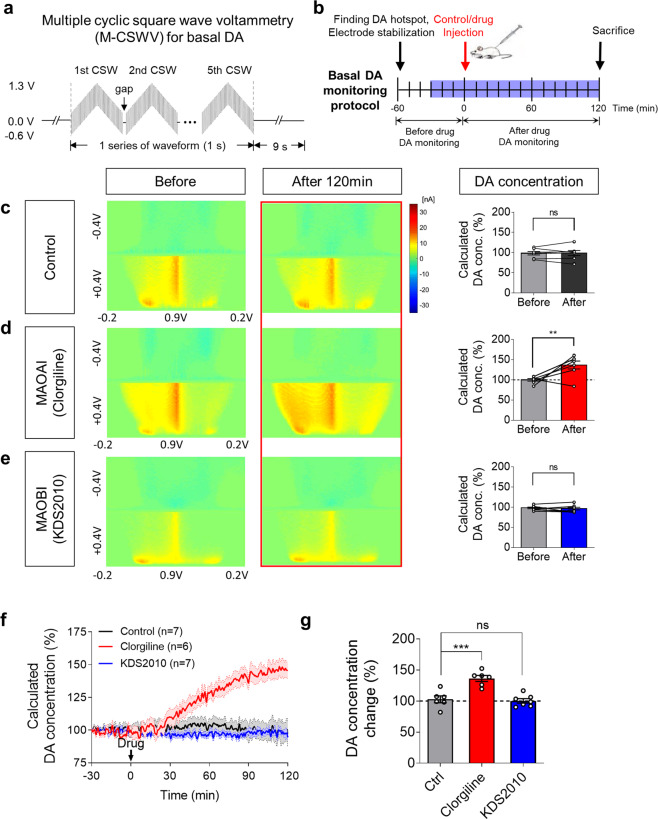
Tonic DA level increased during MAO-A inhibition while MAO-B shows no change in dorsal striatum monitored with MCSWV. **a** Schematic design of a single cyclic square waveform (CSW) and multiple trains of 5 cyclic square wave voltammetry (M-CSWV) waveform. **b** Experimental schedule for tonic DA monitoring. **c**–**e** The representative changes in tonic DA level monitored by M-CSWV in response to treatment with saline control, MAO-A inhibitor (clorgiline, 10 mg kg^−1^), and MAO-B inhibitor (KDS2010, 10 mg kg^−1^) (*N* = 7, 6, and 7). Representative pseudo-color plots demonstrate the tonic DA level in response to M-CSWV before (left) and after 120 mins (middle) from drug administration. The changes in tonic DA concentration changes were quantified (right). **f** Quantification of the time-dependent changes in tonic DA concentrations. **g** Summary bar graph of calculated tonic DA concentrations 120 mins after the treatment of clorgiline and KDS2010 (Same data from **c**–**e**). Data represent mean ± SEM. **P < 0.01; ***P < 0.001; ns, non-significant by paired Student’s t-test (**c–e**) or one-way ANOVA with Tukey’s multiple comparison test (**g**).

In addition to the phasic DA level, we asked whether MAO-A, but not MAO-B, is involved in the maintenance of the basal DA level. Due to the unstable nature of the background currents inherent in the FSCV technique, we should subtract the background signals, which limit the measurement of changes in basal DA levels. Therefore, to measure basal DA levels in the striatum, we utilized in vivo M-CSWV techniques. This technique was recently developed and validated for measuring the changes in basal extracellular DA levels with more extended temporal resolution (10 s) than other basal level detection techniques reported in our previous study^33^. The M-CSWV consisted of a square-wave oscillation superimposed on a symmetric staircase waveform that was applied consecutively multiple times (Fig. 3a, b). The basal DA level, indicated by oxidation peaks seen in red in the region of interest marked with a circle in the range of 0.2 V to 0.45 V, was not altered by the intraperitoneal injection of saline for 120 mins (Fig. 3c). MAO-A inhibition by clorgiline (10 mg kg^−1^) caused a significant increase in the basal DA level after 120 min (147.29 ± 6.39%), whereas MAO-B inhibition by KDS2010 or selegiline (10 mg kg^−1^) did not (97.64 ± 2.91%) (Fig. 3d–g and Supplementary Fig. 1g–i, 3). The real-time change in basal DA could be observed with M-CSWV recordings, which are shown in the corresponding time-dependent graph (Fig. 3f). Only the clorgiline-injected group showed a significant overall increase in basal DA concentration during the 120-min recording compared with the saline-injected, KDS2010-injected, and even selegiline-injected groups (Fig. 3g and Supplementary Fig. 1g–i). These findings indicate that MAO-A inhibition elevates not only phasic DA release but also basal DA levels in the striatum. Taken together, the evidence shows that the inhibition of MAO-A hinders DA degradation, which affects the amount of phasic DA release, the rate of DA uptake, and the basal DA level, while the inhibition of MAO-B does not change DA levels in the striatum.

### MAO-B, but not MAO-A, accounts for tonic GABA current

**Fig. 4:**
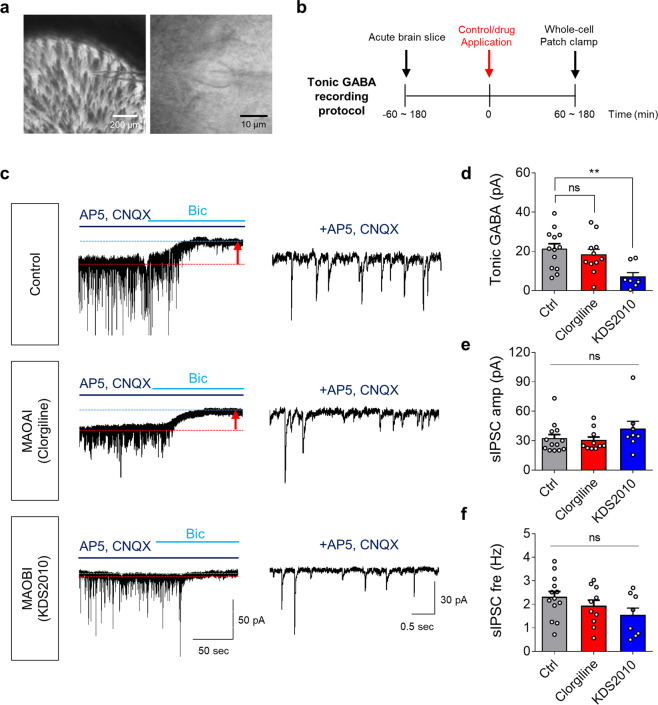
MAO-B, but not MAO-A, is responsible for tonic GABA current in the striatum. **a** Representative image of whole-cell patch-clamp of a neuron in the dorsal striatum. **b** Timeline of whole-cell patch-clamp. **c** Representative traces of tonic GABA recording (left) and sIPSC currents. **d** Quantification of tonic GABA currents (*N* = 5 mice for each group). Tonic GABA current is significantly reduced by MAO-B inhibition (KDS2010), but not by MAO-A inhibition (clorgiline). **e** Quantification of sIPSC amplitudes. **f** Quantification of sIPSC frequency. Data represent mean ± SEM. **P < 0.01; ns, non-significant by Kruskal-Wallis test with Dunn’s multiple comparison test (**d**, **e**) or Brown-Forsythe and Welch ANOVA test with Dunnett’s multiple comparison test (**f**).

We have demonstrated that the inhibition of MAO-B does not affect either phasic or basal DA levels in rodents, which is inconsistent with the traditional belief. Therefore, to explain why MAO-B inhibitors produce symptomatic relief in PD patients, we examined another physiological role of MAO-B. We recently demonstrated that striatal MAO-B is physiologically important for astrocytic GABA synthesis, which is the main cause of tonic inhibition of neighboring neurons^27^. However, whether MAO-A, in addition to MAO-B, participates in tonic inhibition in striatal neurons has not been rigorously tested. Therefore, we performed a whole-cell patch-clamp recording of striatal neurons to record a tonic inhibition current in the presence of MAO-A and MAO-B inhibitors in rats (Fig. 4a, b). We found that the bicuculline (a GABA_A_ receptor antagonist)-sensitive tonic inhibition current of a striatal neuron was ~20 pA. While MAO-A inhibition by 100 nM clorgiline treatment did not affect the tonic current, MAO-B inhibition by 100 nM KDS2010 or selegiline treatment significantly reduced the tonic current by ~70% (Fig. 4c, d and Supplementary Fig. 1j–l). On the other hand, there was no significant difference in the amplitude and frequency of spontaneous inhibitory postsynaptic currents across the groups (Fig. 4c, e, f and Supplementary Fig. 1m, n), indicating that GABAergic synaptic transmission was not altered by either MAO-A or MAO-B inhibition. These findings indicate that only MAO-B, but not MAO-A, is critical for astrocytic GABA-mediated tonic inhibition and that neither MAO-A nor MAO-B participates in neuronal GABA-mediated phasic inhibition.

## Discussion

**Fig. 5:**
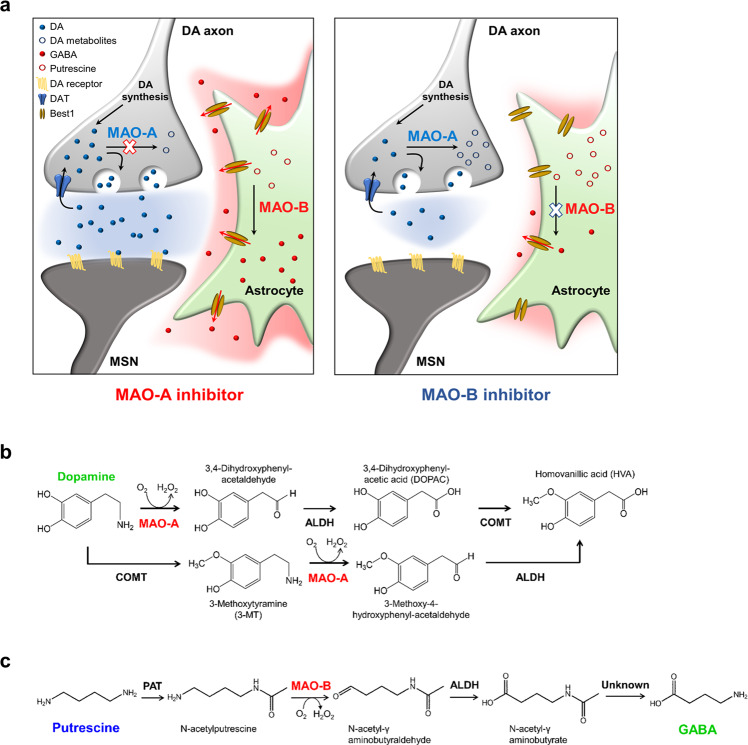
Suggested roles of MAO-A and MAO-B in the striatum. **a** Schematic diagram of the effect of MAO-A inhibitor and MAO-B inhibitor in the striatum. MAO-A in neurons is responsible for DA degradation, whereas MAO-B in astrocytes has a role in GABA synthesis from putrescine. **b** MAO-A dependent dopamine degradation pathway in neurons. **c** MAO-B dependent GABA synthesis pathway in astrocytes.

In this study, we demonstrated that MAO-A and MAO-B have differential roles in regulating phasic/tonic DA and tonic GABA levels, respectively. In vivo electrochemical monitoring of DA currents using FSCV and M-CSWV and ex vivo DA imaging using a GRAB_DA2m_ sensor revealed that the acute pharmacological inhibition of MAO-B by KDS2010 and selegiline did not affect either phasic or basal DA levels, which was inconsistent with the traditional belief. On the other hand, MAO-A inhibition by clorgiline significantly increased the levels of phasic and basal DA, which could be attributed to a blockade of DA metabolism. Moreover, ex vivo whole-cell patch-clamp experiments revealed that MAO-B plays an important role in astrocytic GABA-mediated tonic inhibition, whereas MAO-A does not. Taken together, our findings provide conclusive in vivo evidence for resolving the controversy on the role of MAO-B in DA metabolism, which has continued over several decades. (Fig. 5).

The contribution of MAO to DA degradation was reported over six decades ago^41^. The development of specific inhibitors against MAO-A (clorgiline) and MAO-B (selegiline) in the 1960s made it possible to investigate which is the major enzyme for DA metabolism between the two isoenzymes. Through intense investigations on this issue in the 1970s^13–16^, both MAO-A and MAO-B were reported to be responsible for DA metabolism in the human brain, but the contributions varied among brain regions^17^. Moreover, several clinical studies have demonstrated that MAO-B inhibitors show efficacy in the treatment of DA-deficient states, such as PD^42,43^, which supports the idea that MAO-B is critically involved in DA degradation. However, direct evidence has been lacking. Human studies from postmortem brain homogenates have provided only indirect evidence.

On the other hand, several in vivo microdialysis and high-performance liquid chromatography (HPLC) studies in live rodents cast doubt on the previous belief in the 1990s that MAO-B was the key enzyme for DA degradation by demonstrating that the pharmacological inhibition of MAO-B minimally alters the basal DA concentration^5,18–21,44^. Although microdialysis and HPLC methods have advantages such as high chemical specificity^45^ and sensitivity^46–48^ for detecting DA, they have several critical drawbacks. First, the insertion of a microdialysis probe (~200–300 μm) can cause significant tissue damage^49,50^, leading to perturbation of the physiological neuronal function^51,52^. Second, it is difficult to study the mechanisms of fast synaptic release and the reuptake of DA with microdialysis, both of which critically affect the extracellular DA concentration^53,54^. These critical drawbacks have hindered widespread that MAO-B is less responsible for DA degradation, which is against the traditional belief. To overcome these drawbacks, we used two advanced electrochemical techniques, FSCV and M-CSWV, to investigate the phasic and tonic DA levels in the extracellular space, respectively. FSCV is a well-established electrochemical technique to measure the phasic changes of electroactive neurotransmitters, such as DA, by applying a fast potential scan (>10 Hz) into a carbon-fiber microelectrode (CFME)^55,56^. In addition, the diameter of a CFME is ~10 μm, which permits greater spatial resolution and less tissue damage^57^. M-CSWV is a recently developed voltammetric technique to measure tonic DA concentration by using cyclic square wave voltammetric waveforms in conjunction with a delayed holding potential period to control DA adsorption to the CFME surface. M-CSWV demonstrates high in vivo selectivity, high sensitivity (a detection limit of ~0.17 nM), and 10 s (0.1 Hz) temporal resolution^33^. These advantages of FSCV and M-CSWV allowed us to measure the exact phasic and tonic DA changes upon treatment with MAO-A and MAO-B inhibitors. In addition to these electrochemical approaches, our ex vivo DA imaging with a newly developed DA sensor, GRAB_DA2m_, which yields a high spatiotemporal resolution, high specificity, and high sensitivity, demonstrated consistent results with the findings from FSCV experiments. Taken together, our consistent findings across the two different approaches provide strong evidence that MAO-A, but not MAO-B, is engaged in DA degradation.

Several previous studies have suggested that a possible mode of action of MAO-B inhibitors in PD patients involves the stabilization of mitochondria and the induction of the antiapoptotic Bcl-2 protein family and neurotrophic factors^58–60^. In addition, MAO-B inhibitors have been suggested to hinder DA metabolism in serotonergic neurons, specifically in the brains of PD patients, based on a previous study demonstrating that L-DOPA is mostly metabolized and released by serotonergic neurons that express MAO-B in the DA-depleted striatum^61^. In addition, we report another exclusive role of MAO-B in tonic GABA inhibition of striatal neurons, which is not mediated by MAO-A. We recently reported that MAO-B-mediated tonic GABA inhibition in the SNpc is critical for DA neuronal dysfunction and parkinsonian motor symptoms in various animal models of PD^24^. Our findings suggest that the therapeutic effect of MAO-B inhibitors could be attributed to blocking astrocytic GABA synthesis rather than to blocking DA degradation. These findings may further resolve the conflict between the minimal role of MAO-B in DA metabolism and the therapeutic effect of MAO-B inhibitors in PD.

In addition to DA, other transmitters such as epinephrine, norepinephrine, and serotonin could be metabolized by MAO in the brain. These transmitters are well documented to be mainly metabolized by MAO-A rather than MAO-B due to the substrate preference of the enzyme. Moreover, transporters for these transmitters are mainly expressed in neurons expressing MAO-A but not in astrocytes expressing MAO-B. Therefore, MAO-A, not MAO-B, might be responsible for degrading other catecholamines regardless of the brain region.

How should we resolve the discrepancy between the results from in vitro and in vivo studies regarding MAO-B? Several in vitro studies demonstrated that MAO-B also utilizes DA as a substrate in both rats (Km = ~340 μM)^62^ and humans (Km = ~210–230 μM)^17^. Why is MAO-B not involved in DA degradation in the striatum in vivo even though MAO-B is capable of DA degradation in vitro? Because the level of DAT expression in striatal astrocytes is very low, the released DA is minimally taken up by astrocytes, in which MAO-B is mainly expressed under physiological conditions^23^. On the other hand, DA neurons, in which MAO-A is mainly expressed, are predominantly responsible for the reuptake of synaptically released DA through DAT in the dorsal striatum^40,63^. In addition to the striatum, DAT is expressed mainly in neurons but not in astrocytes in most brain regions, including the substantia nigra, nucleus accumbens, and cortical areas^63–65^. Therefore, regardless of the ability of MAO-B to metabolize DA, the amount of astrocytic MAO-B-mediated DA degradation is negligible in various regions, including the striatum, in the healthy brain.

In conclusion, our findings demonstrate that MAO-A, but not MAO-B, is mainly responsible for regulating the phasic and tonic DA levels in the striatum by mediating DA degradation. MAO-B, but not MAO-A, is mainly responsible for the tonic inhibition of striatal neurons by mediating astrocytic GABA synthesis. Our results are important to differentiate the obscure roles of MAO-A and MAO-B in DA metabolism and GABA synthesis. Our study re-establishes the roles of MAO-A and MAO-B under physiological conditions: MAO-A in DA degradation and MAO-B in tonic GABA synthesis.

## Supplementary information

Supplementary information

## References

[1] Shih JC, Chen K, Ridd MJ (1999). Role of MAO A and B in neurotransmitter metabolism and behavior. Pol. J. Pharm..

[2] Cai Z (2014). Monoamine oxidase inhibitors: promising therapeutic agents for Alzheimer’s disease (Review). Mol. Med. Rep..

[3] Bortolato M, Chen K, Shih JC (2008). Monoamine oxidase inactivation: from pathophysiology to therapeutics. Adv. Drug Deliv. Rev..

[4] Edmondson DE, Binda C, Mattevi A (2007). Structural insights into the mechanism of amine oxidation by monoamine oxidases A and B. Arch. Biochem. Biophys..

[5] Brannan T, Prikhojan A, Martinez-Tica J, Yahr MD (1995). In vivo comparison of the effects of inhibition of MAO-A versus MAO-B on striatal L-DOPA and dopamine metabolism. J. Neural Transm. Park Dis. Dement. Sect..

[6] Westlund KN, Denney RM, Rose RM, Abell CW (1988). Localization of distinct monoamine oxidase A and monoamine oxidase B cell populations in human brainstem. Neuroscience.

[7] Fagervall I, Ross SB (1986). A and B forms of monoamine oxidase within the monoaminergic neurons of the rat brain. J. Neurochem..

[8] Thorpe LW, Westlund KN, Kochersperger LM, Abell CW, Denney RM (1987). Immunocytochemical localization of monoamine oxidases A and B in human peripheral tissues and brain. J. Histochem. Cytochem..

[9] Finberg JP, Rabey JM (2016). Inhibitors of MAO-A and MAO-B in psychiatry and neurology. Front. Pharm..

[10] Youdim MBH, Edmondson D, Tipton KF (2006). The therapeutic potential of monoamine oxidase inhibitors. Nat. Rev. Neurosci..

[11] Park JH (2019). Newly developed reversible MAO-B inhibitor circumvents the shortcomings of irreversible inhibitors in Alzheimer’s disease. Sci. Adv..

[12] Caccia C (2006). Safinamide: from molecular targets to a new anti-Parkinson drug. Neurology.

[13] Roth JA, Feor K (1978). Deamination of dopamine and its 3-O-methylated derivative by human brain monoamine oxidase. Biochem. Pharm..

[14] Glover V, Elsworth JD, Sandler M (1980). Dopamine oxidation and its inhibition by (-)-deprenyl in man. J. Neural Transm. Suppl..

[15] Tipton KF, Houslay MD, Garrett NJ (1973). Allotopic properties of human brain monoamine oxidase. Nat. N. Biol..

[16] Glover V, Sandler M, Owen F, Riley GJ (1977). Dopamine is a monoamine oxidase B substrate in man. Nature.

[17] O’Carroll AM, Fowler CJ, Phillips JP, Tobbia I, Tipton KF (1983). [The deamination of dopamine by human brain monoamine oxidase. Specificity for the two enzyme forms in seven brain regions]. Naunyn Schmiedebergs Arch. Pharm..

[18] Fornai F (1999). Striatal dopamine metabolism in monoamine oxidase B-deficient mice: a brain dialysis study. J. Neurochem..

[19] Butcher SP, Fairbrother IS, Kelly JS, Arbuthnott GW (1990). Effects of selective monoamine oxidase inhibitors on the in vivo release and metabolism of dopamine in the rat striatum. J. Neurochem..

[20] Scarr E, Wingerchuk DM, Juorio AV, Paterson IA (1994). The effects of monoamine oxidase B inhibition on dopamine metabolism in rats with nigro-striatal lesions. Neurochem. Res..

[21] Paterson IA, Juorio AV, Berry MD, Zhu MY (1991). Inhibition of monoamine oxidase-B by (-)-deprenyl potentiates neuronal responses to dopamine agonists but does not inhibit dopamine catabolism in the rat striatum. J. Pharm. Exp. Ther..

[22] Hashemi P (2012). Brain dopamine and serotonin differ in regulation and its consequences. Proc. Natl Acad. Sci. USA.

[23] Sader-Mazbar O, Loboda Y, Rabey MJ, Finberg JP (2013). Increased L-DOPA-derived dopamine following selective MAO-A or -B inhibition in rat striatum depleted of dopaminergic and serotonergic innervation. Br. J. Pharm..

[24] Heo JY (2020). Aberrant tonic inhibition of dopaminergic neuronal activity causes motor symptoms in animal models of Parkinson’s disease. Curr. Biol..

[25] Jo S (2014). GABA from reactive astrocytes impairs memory in mouse models of Alzheimer’s disease. Nat. Med..

[26] Nam MH (2020). Excessive astrocytic GABA causes cortical hypometabolism and impedes functional recovery after subcortical stroke. Cell Rep..

[27] Yoon BE (2014). Glial GABA, synthesized by monoamine oxidase B, mediates tonic inhibition. J. Physiol..

[28] Lee S (2010). Channel-mediated tonic GABA release from glia. Science.

[29] Kwak H (2020). Astrocytes control sensory acuity via tonic inhibition in the thalamus. Neuron.

[30] Chun H (2020). Severe reactive astrocytes precipitate pathological hallmarks of Alzheimer’s disease via H2O2(-) production. Nat. Neurosci..

[31] Chun H (2018). Astrocytic proBDNF and tonic GABA distinguish active versus reactive astrocytes in hippocampus. Exp. Neurobiol..

[32] Venton BJ, Cao Q (2020). Fundamentals of fast-scan cyclic voltammetry for dopamine detection. Analyst.

[33] Oh Y (2018). Tracking tonic dopamine levels in vivo using multiple cyclic square wave voltammetry. Biosens. Bioelectron..

[34] Sun F (2020). Next-generation GRAB sensors for monitoring dopaminergic activity in vivo. Nat. Methods.

[35] Paxinos, G. & Watson, C. *The Rat Brain in Stereotaxic Coordinates: Hard Cover Edition*. (Elsevier, 2006).

[36] Vreeland RF (2015). Biocompatible PEDOT:Nafion composite electrode coatings for selective detection of neurotransmitters in vivo. Anal. Chem..

[37] Franklin, K. & Paxinos, G. *Paxinos and Franklin’s the Mouse Brain in Stereotaxic Coordinates*. (Elsevier, 2019).

[38] Grace AA (2000). The tonic/phasic model of dopamine system regulation and its implications for understanding alcohol and psychostimulant craving. Addiction.

[39] Klanker M, Feenstra M, Willuhn I, Denys D (2017). Deep brain stimulation of the medial forebrain bundle elevates striatal dopamine concentration without affecting spontaneous or reward-induced phasic release. Neuroscience.

[40] Asanuma, M., Miyazaki, I., Murakami, S., Diaz-Corrales, F. J. & Ogawa, N. Striatal Astrocytes Act as a Reservoir for L-DOPA. *PLoS ONE* **9**, ARTN e106362 10.1371/journal.pone.0106362 (2014). 10.1371/journal.pone.0106362 PMC4154692 25188235

[41] Weiner N (1960). Substrate specificity of brain amine oxidase of several mammals. Arch. Biochem Biophys..

[42] Finberg JPM (2019). Inhibitors of MAO-B and COMT: their effects on brain dopamine levels and uses in Parkinson’s disease. J. Neural Transm.

[43] Tong J (2017). Brain monoamine oxidase B and A in human parkinsonian dopamine deficiency disorders. Brain.

[44] Lamensdorf I, Youdim MB, Finberg JP (1996). Effect of long-term treatment with selective monoamine oxidase A and B inhibitors on dopamine release from rat striatum in vivo. J. Neurochem.

[45] Rusheen, A. E. et al. Evaluation of electrochemical methods for tonic dopamine detection in vivo. *Trac.-Trend Anal. Chem.* **132**, ARTN 116049 10.1016/j.trac.2020.116049 (2020). 10.1016/j.trac.2020.116049 PMC7885180 33597790

[46] Heidbreder CA (2001). Development and application of a sensitive high performance ion-exchange chromatography method for the simultaneous measurement of dopamine, 5-hydroxytryptamine and norepinephrine in microdialysates from the rat brain. J. Neurosci. Methods.

[47] Gu H, Varner EL, Groskreutz SR, Michael AC, Weber SG (2015). In vivo monitoring of dopamine by microdialysis with 1 min temporal resolution using online capillary liquid chromatography with electrochemical detection. Anal. Chem..

[48] Yoshitake T, Kehr J, Todoroki K, Nohta H, Yamaguchi M (2006). Derivatization chemistries for determination of serotonin, norepinephrine and dopamine in brain microdialysis samples by liquid chromatography with fluorescence detection. Biomed. Chromatogr..

[49] Clapp-Lilly KL (1999). An ultrastructural analysis of tissue surrounding a microdialysis probe. J. Neurosci. Methods.

[50] Szarowski DH (2003). Brain responses to micro-machined silicon devices. Brain Res..

[51] Di Chiara G (1993). Stimulation of dopamine transmission in the dorsal caudate nucleus by pargyline as demonstrated by dopamine and acetylcholine microdialysis and Fos immunohistochemistry. Neuroscience.

[52] Blaha CD, Coury A, Phillips AG (1996). Does monoamine oxidase inhibition by pargyline increase extracellular dopamine concentrations in the striatum?. Neuroscience.

[53] Yang H (2000). A theoretical description of microdialysis with mass transport coupled to chemical events. Anal. Chem..

[54] Chen KC, Budygin EA (2007). Extracting the basal extracellular dopamine concentrations from the evoked responses: re-analysis of the dopamine kinetics. J. Neurosci. Methods.

[55] Heien ML, Johnson MA, Wightman RM (2004). Resolving neurotransmitters detected by fast-scan cyclic voltammetry. Anal. Chem..

[56] Jaquins-Gerstl A, Michael AC (2015). A review of the effects of FSCV and microdialysis measurements on dopamine release in the surrounding tissue. Analyst.

[57] Peters JL, Miner LH, Michael AC, Sesack SR (2004). Ultrastructure at carbon fiber microelectrode implantation sites after acute voltammetric measurements in the striatum of anesthetized rats. J. Neurosci. Methods.

[58] Naoi M, Maruyama W (2010). Monoamine oxidase inhibitors as neuroprotective agents in age-dependent neurodegenerative disorders. Curr. Pharm. Des..

[59] Weinreb O, Amit T, Bar-Am O, Youdim MBH (2010). Rasagiline: a novel anti-Parkinsonian monoamine oxidase-B inhibitor with neuroprotective activity. Prog. Neurobiol..

[60] Szoko E, Tabi T, Riederer P, Vecsei L, Magyar K (2018). Pharmacological aspects of the neuroprotective effects of irreversible MAO-B inhibitors, selegiline and rasagiline, in Parkinson’s disease. J. Neural Transm..

[61] Navailles S, Bioulac B, Gross C, De Deurwaerdere P (2010). Serotonergic neurons mediate ectopic release of dopamine induced by L-DOPA in a rat model of Parkinson’s disease. Neurobiol. Dis..

[62] Fowler CJ, Benedetti MS (1983). The metabolism of dopamine by both forms of monoamine oxidase in the rat brain and its inhibition by cimoxatone. J. Neurochem..

[63] Sesack SR, Hawrylak VA, Matus C, Guido MA, Levey AI (1998). Dopamine axon varicosities in the prelimbic division of the rat prefrontal cortex exhibit sparse immunoreactivity for the dopamine transporter. J. Neurosci..

[64] Nirenberg MJ, Vaughan RA, Uhl GR, Kuhar MJ, Pickel VM (1996). The dopamine transporter is localized to dendritic and axonal plasma membranes of nigrostriatal dopaminergic neurons. J. Neurosci..

[65] Nirenberg MJ (1997). The dopamine transporter: comparative ultrastructure of dopaminergic axons in limbic and motor compartments of the nucleus accumbens. J. Neurosci..

