# Supplementary material for: Redefining differential roles of MAO-A in dopamine degradation and MAO-B in tonic GABA synthesis
Source: Exp Mol Med. 2021 Jul 9;53(7):1148–58. doi: 10.1038/s12276-021-00646-3 (PMC8333267; doi:10.1038/s12276-021-00646-3)
Supplement: Supplementary file 1 — Supplementary information [file 12276_2021_646_MOESM1_ESM.pdf]

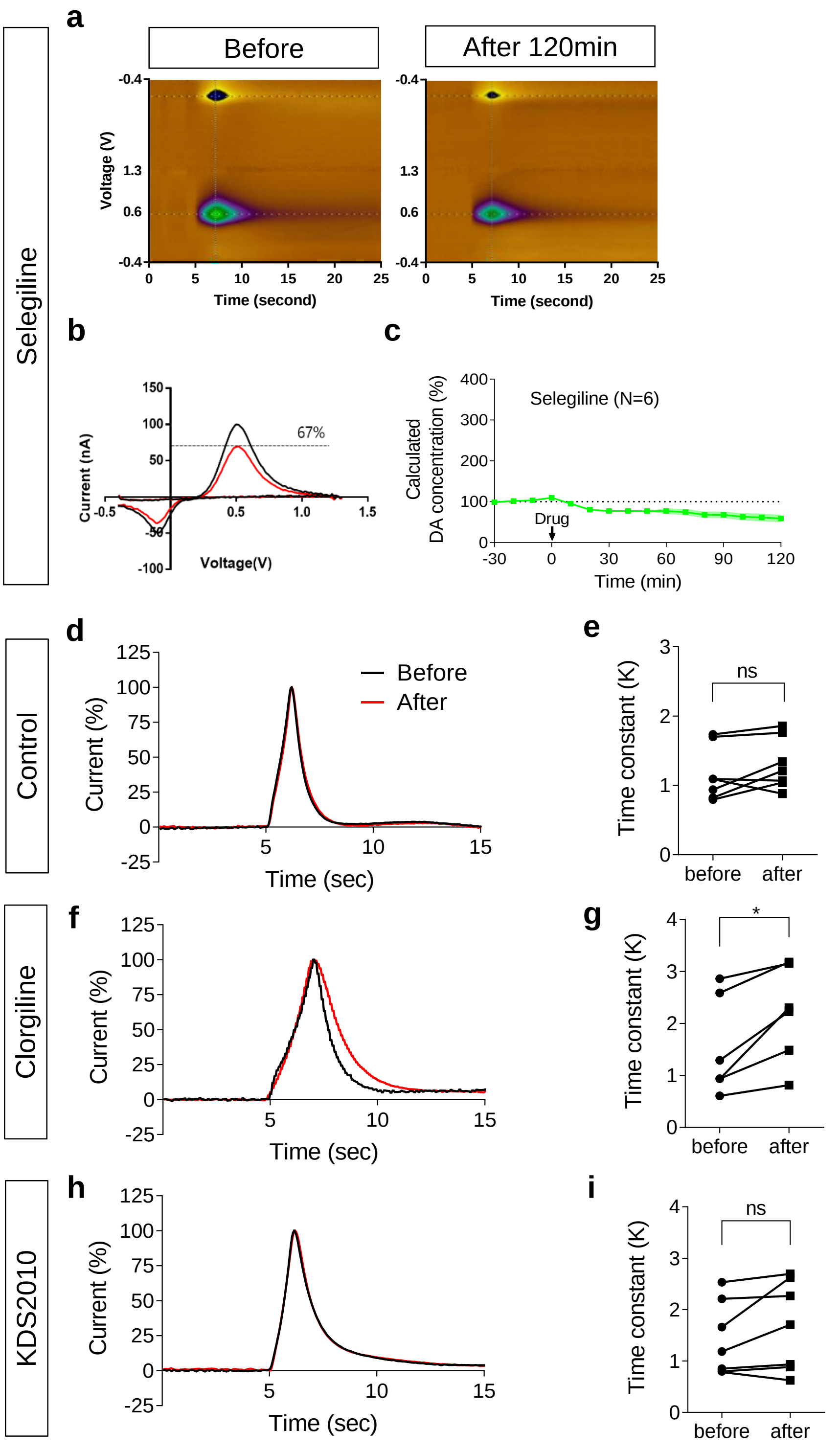

Supplementary Fig. 1. Effect of selegiline, another MAO-B inhibitor, in phasic dopamine release and measurement of time constant with MAO-A and MAO-B inhibitor in rat striatum.

a. The representative phasic dopamine changes in the striatum detected by FSCV in response to treatment with MAO-B inhibitor (selegiline, 10 mg kg^{-1}). Pseudo-color plots indicate the phasic dopamine responses before (left) and after 120 mins (middle) from selegiline administration. b. Comparison of phasic dopamine release between before and 120 after selegiline treatment. c. Time-dependent change in the peak DA current (n=6). d, f, h. Time-series plot of each evoked DA response was extracted at the dopamine oxidation potential from FSCV recording. The black line and red line show the concentration change before and after drug injection respectively. e, g, i. Quantification of time constant K before and after drug injection. Time constant K was increased by MAO-A inhibition (clorgiline) but not by MAO-B inhibition (KDS2010). *P < 0.01; ns, non-significant by two-tailed Student's paired t-test.

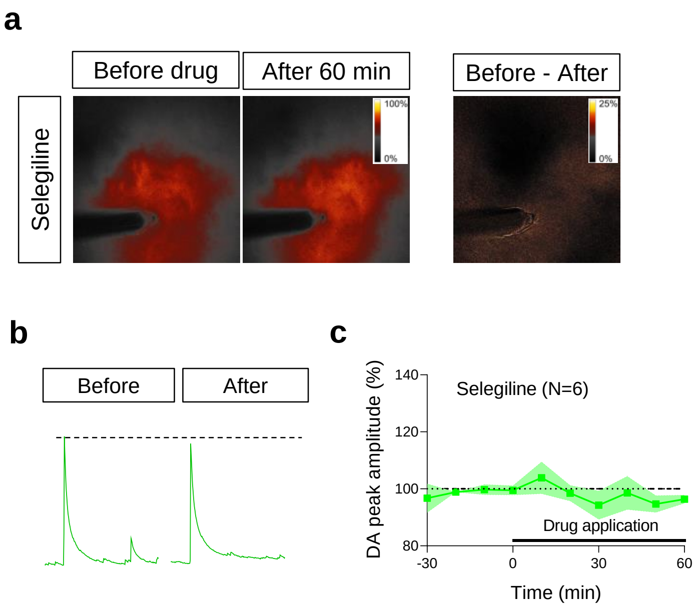

Supplementary Fig. 2. Application of selegiline, another MAO-B inhibitor, does not change the level of phasic dopamine release in mouse striatum.

a. Representative images of phasic DA release level after 60 min of selegiline treatment. b. Example DA trace from a. Dotted line indicates the level of phasic DA release before drug treatment. c. Time-dependent changes in DA peak amplitudes before and during drug treatment (N= 6). Phasic dopamine release is not changed by selegiline treatment.

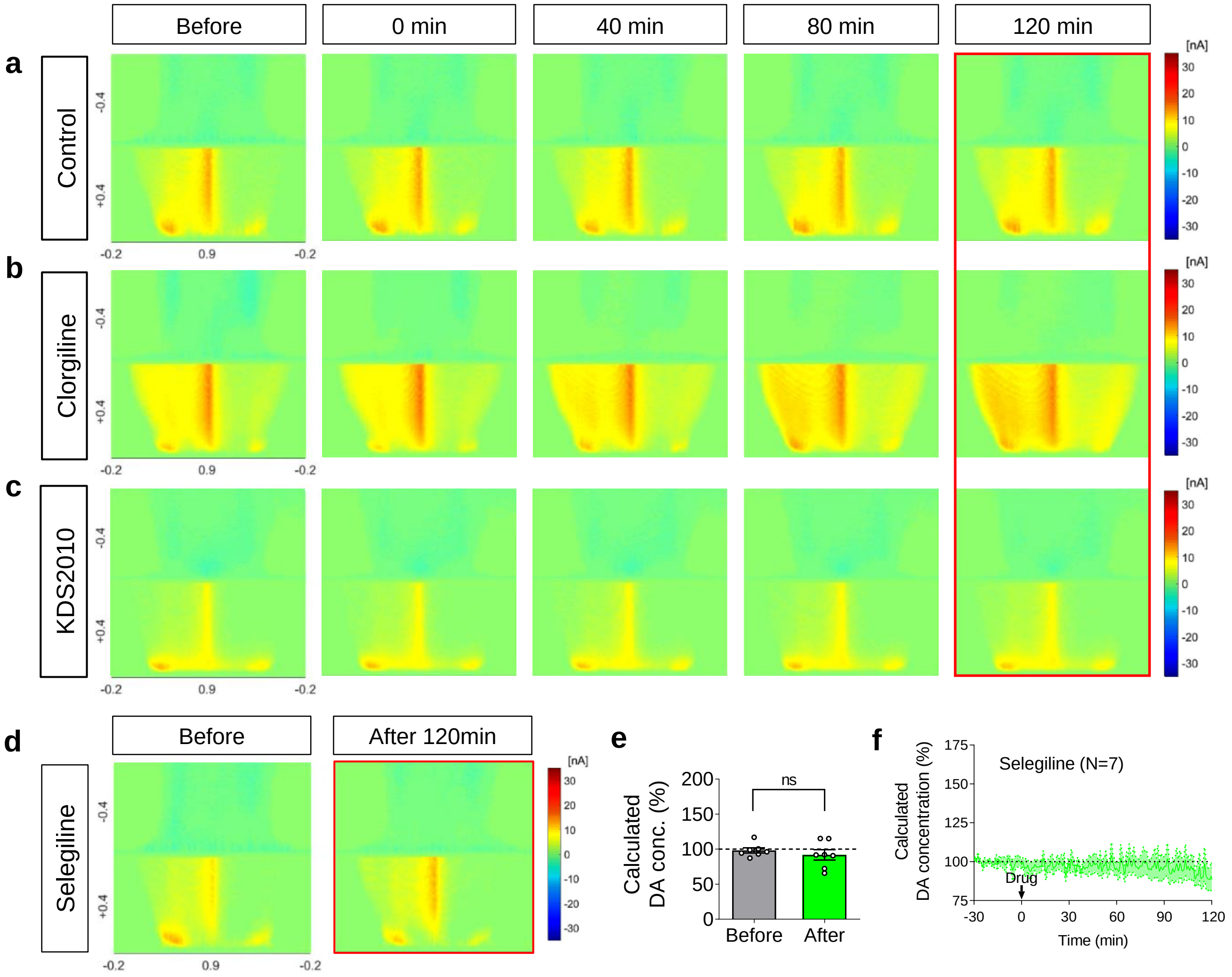

Supplementary Fig. 3. Time-course recordings of M-CSWV show increased tonic DA level during MAO-A inhibition but no change from MAO-B in striatum.

a, b, c. Time-course recordings of M-CSWV show that MAO-A inhibition (clorgiline) markedly increased the tonic DA level, while MAO-B inhibition (KDS2010) did not. . d. The representative changes in tonic DA level monitored by M-CSWV in response to treatment with selegiline (10 mg kg−1) (N = 7). Representative pseudo-color plots demonstrate the tonic DA level in response to M-CSWV before (left) and after 120 mins (right) from drug administration. e. The changes in tonic DA concentration changes were quantified. f. Quantification of the time-dependent changes in tonic DA concentrations.

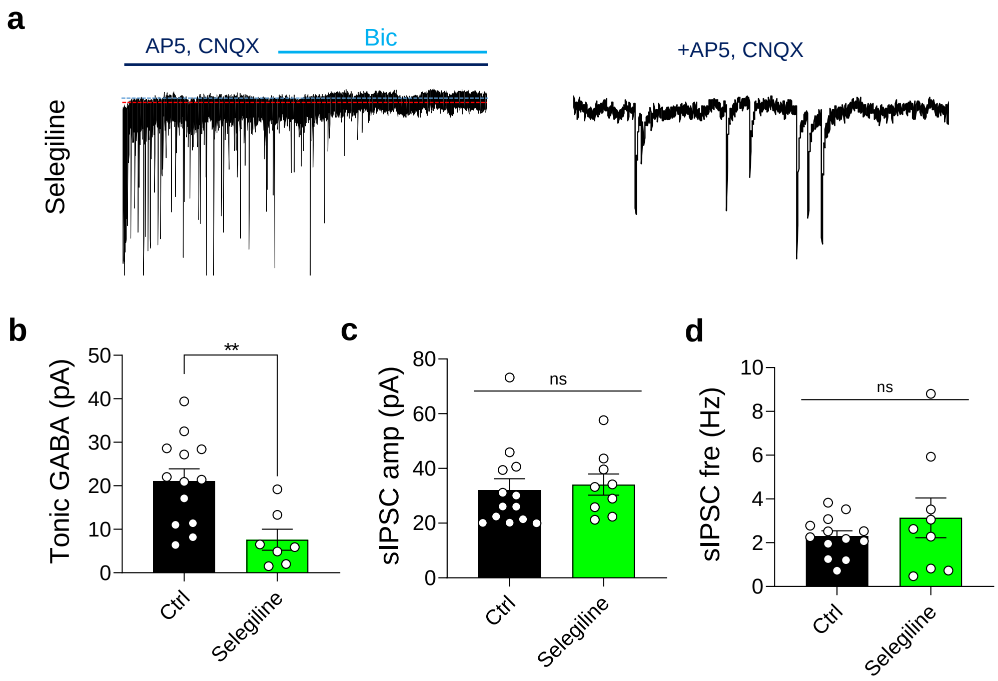

Supplementary Fig. 4. Selegiline, another MAO-B inhibitor, decreases tonic GABA current in striatum.

a. Representative traces of tonic GABA recording (left) and sIPSC currents (right). b. Quantification of tonic GABA currents (N = 5 mice for ctrl and selegiline group). Tonic GABA current is significantly reduced by selegiline treatment. c-d. No change of sIPSC amplitude and frequency after selegiline treatment. Data represent mean ± SEM. **P < 0.01; ns, non-significant.
